# Predicting outcomes in critically ill patients in a resource-poor setting: the Rwanda Mortality Probability Model

**DOI:** 10.1186/cc14625

**Published:** 2015-03-16

**Authors:** T Twagirumugabe, E Riviello, R Fowler, V Novack, A Mueller, W Kiviri, V Banner-Goodspeed, D Talmor

**Affiliations:** 1 University of Rwanda, Kigali, Rwanda; 2 Beth Israel Deaconess Medical Center, Boston, MA, USA; 3 University of Toronto, ON, Canada; 4 Ben-Gurion University of the Negev, Beer Sheva, Israel

## Introduction

ICU mortality prediction models provide robust tools for research and benchmarking in the developed world, but an ICU mortality prediction model has not been validated in a resource-poor setting. We sought to validate the Mortality Probability Admission Model, version III (MPMo-III) in two public ICUs in Rwanda and to develop a simplified Rwanda Mortality Probability Model (R-MPM) for use in developing countries.

## Methods

We prospectively collected data on 339 adult patients admitted to two ICUs in Rwanda between August 2013 and July 2014. We described demographic and presenting characteristics and outcomes. We assessed the discrimination and calibration of the MPMo-III model. Using stepwise selection, we then developed a new logistic model for mortality prediction, the R-MPM.

## Results

Patient median age was 34 (IQR 26 to 49) years; 48.7% were male. Mortality was 50.3%. The variables most predictive of mortality in univariate analyses were: age, sepsis within 24 hours of ICU admission, hypotension or shock at ICU admission, Glasgow Coma Scale score at ICU admission, and heart rate (beats per minute) at ICU admission. Using these five variables, the R-MPM predicted mortality with area under the curve of 0.829 and Hosmer-Lemeshow chi-square statistic of 8.881. The MPMo-III predicted mortality with area under the curve of 0.720 and Hosmer-Lemeshow chi-square statistic of 16.391, indicating that the predictive risk scores of the MPMo-III were not well calibrated to the Rwandan data.

## Conclusion

The MPMo-III had modest risk prediction capacity in a population of Rwandan ICU patients. The R-MPM is an alternative severity score with fewer and more accessible variables that provides better predictive power. This model needs to be validated in other ICUs.

